# Delineating the role of eIF2α in retinal degeneration

**DOI:** 10.1038/s41419-019-1641-y

**Published:** 2019-05-28

**Authors:** Christopher R. Starr, Marina S. Gorbatyuk

**Affiliations:** Department of Optometry and Vision Science, The University of Alabama at Birmingham, School of Optometry, Birmingham, AL USA

**Keywords:** Apoptosis, Diseases

## Abstract

Activation of the unfolded protein response has been detected in various animal models of retinal degeneration. The PERK branch converges on eIF2α to regulate protein synthesis. We previously reported that diseased retinas produce less protein as they degenerate. We also proposed that the majority of this reduction in protein synthesis may not be due to control of eIF2α. Nevertheless, multiple research groups have reported that modulating eIF2α levels may be a viable strategy in the treatment of neurodegenerative diseases. Here, using two genetic approaches, a systemic *Gadd34* knockout and a photoreceptor conditional *Perk* knockout, to alter p-eIF2α levels in *rd16* mice, we demonstrate not only that degenerating retinas may not use this mechanism to signal for a decline in protein synthesis rates but also that modulation of p-eIF2α levels is insufficient to delay retinal degeneration.

## Introduction

Inherited retinal degeneration (IRD) is a class of retinal dystrophies in which there is currently no cure and few treatment options available. Activation of the unfolded protein response (UPR) has been detected in multiple animal models of retinal degeneration^1–5^. The UPR is a series of signaling events following endoplasmic reticulum (ER) stress and is signaled through three ER – resident membrane proteins, protein kinase R like-endoplasmic reticulum kinase (PERK), inositol requiring enzyme 1 (IRE1) and activating transcription factor 6 (ATF6). PERK signals for a halt in translation following ER stress by phosphorylating eukaryotic initiation factor 2 alpha (eIF2α)^6,7^. This translational attenuation of new protein synthesis aims to reduce the burden on the cell and provides cells with an opportunity to refold incorrectly folded proteins and adapt^7^.

Multiple groups have reported elevated activation of the PERK branch in animal models of retinal degeneration. There are no known roles of eIF2α other than protein synthesis regulation, so it has been assumed that its phosphorylation leads to a substantial attenuation in protein synthesis rates, but no study has functionally and genetically modulated p-eIF2α in degenerating retinas and subsequently assessed retinal protein synthesis rates and photoreceptor survival. In addition, as we have proposed previously^1^, eIF2α may not be the primary point of translational control in degenerating retinas. Those findings make us question what role, if any, eIF2α phosphorylation plays in retinal degeneration.

The phosphorylation of eIF2α by one or more kinases, together with the alternative translational and transcriptional program has been coined the integrated stress response, or ISR^8^. When eIF2α is phosphorylated, eIF2 can no longer participate in translation and protein synthesis rates typically go down; however, translation of certain mRNAs with upstream open reading frames (uORFs) is actually promoted in these conditions^6^. One protein known to be selectively translated following eIF2α phosphorylation is ATF4, a transcription factor that promotes expression of several stress related genes including growth arrest and DNA-damage 34 (GADD34)^9^. GADD34, also known as protein phosphatase 1 regulatory subunit 15A (PPP1R15A), is the stress induced-phosphatase regulatory subunit that recruits protein phosphatase 1 (PP1) and initiates the dephosphorylation of phosphorylated-eIF2α (p-eIF2α)^9–11^. Therefore, GADD34 is a central component of the feedback loop aiming to restore translation following a transient stress. Another important player of PP1 is CreP (PP1R15B), which is responsible for maintaining basal levels of p-eIF2a^12^. Aside from regulating eIF2α phosphorylation, GADD34 has a multitude of other roles including promoting apoptosis^13–16^. Interestingly, a recent study suggested that PP1/GADD34 may act on a plethora of previously unknown targets^13^.

We previously reported that a majority of eIF2α phosphorylation is likely due to PKR-like endoplasmic reticulum kinase (PERK) in *rd16* mice^1^. Although we also revealed that eIF2α may not be the primary means by which degenerating retinas control translation^1^, it is still unknown whether this mechanism plays a role in RD. Recently, multiple research groups have demonstrated that diminishing PERK under chronic ER stress can result in improvement of neuronal function and survival in neurodegenerative diseases^17–20^. While important, these studies have not focused on validation of this therapeutic strategy to restore general protein synthesis, first and have not compared a degree of the restoration in the same animal model modulating other regulatory nodes of translation, such as 4E-BP1/2. Therefore, the mentioned studies demonstrate the gap in knowledge in this field and indicates that the role of p-eIF2a under chronic ER stress needs to be examined carefully. In this study, we aim to delineate whether eIF2α plays a significant role in maintaining protein synthesis under chronic ER stress and therefore, determine if it contributes to progressive retinopathy.

## Results

### Strategy to modulate the eIF2a activity

The ability of eIF2α to regulate protein synthesis and therefore allow the cell to cope with stress depends on its phosphorylation status. Thus, when phosphorylated, eIF2α mediates the binding of the initiator tRNA-Met to the ribosome in a GTP-dependent manner. In order to modulate its activity, we decided to up- and down-regulate its phosphorylation state, which requires either deactivating protein phosphatase (PP1) or the active eIF2α kinase, PERK. To that end, we generated *rd16 Gadd34*^−/−^ and *rd16 Perk*^*f/f*^ iCre75 mice to access protein synthesis and investigate their contribution to translational modulation during chronic activation of the ISR.

### Increasing the p-eIF2a does not further diminish protein synthesis but delays retinal degeneration via GADD34 ablation

**Fig. 1:**
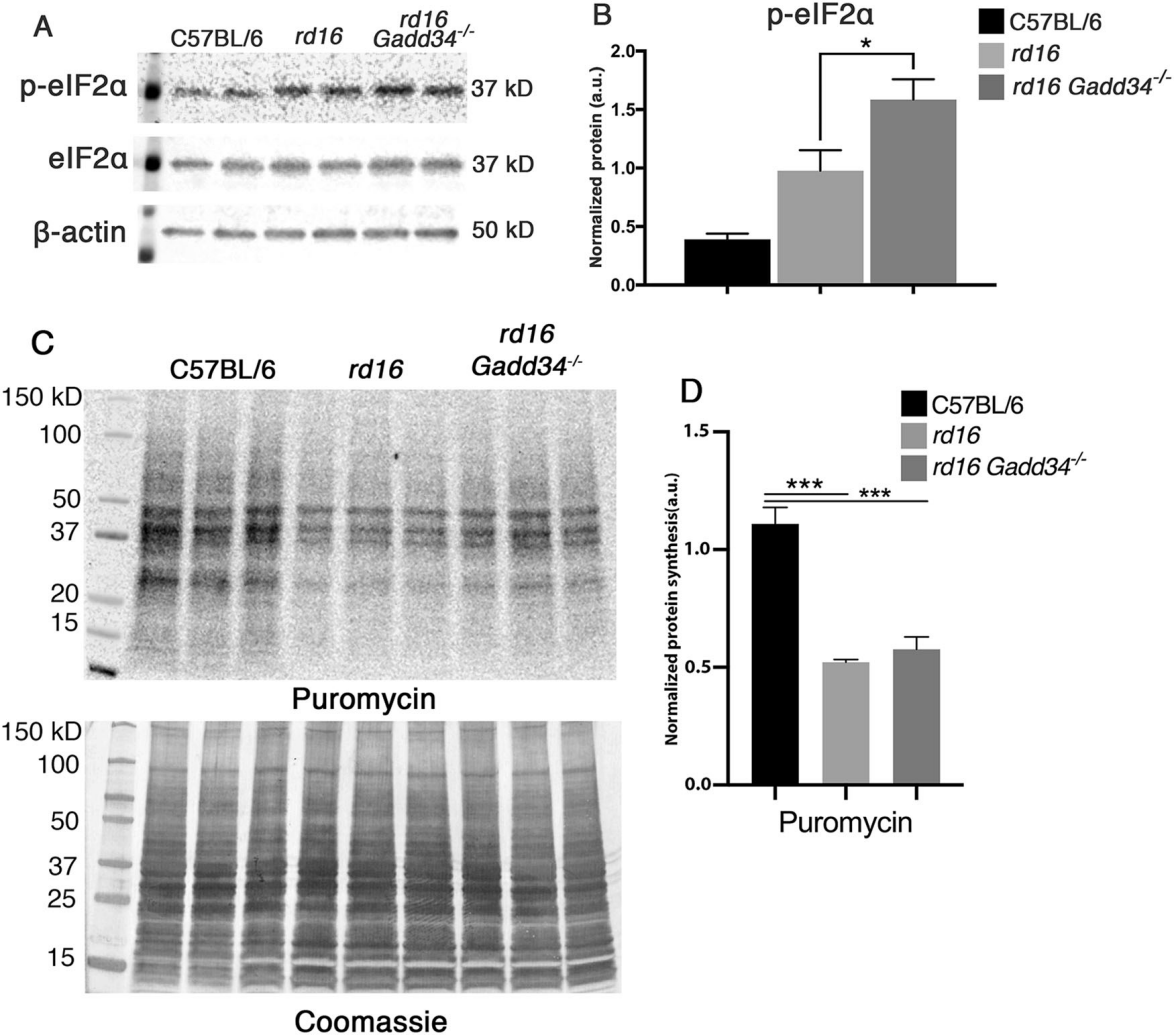
Loss of GADD34 increases p-eIF2α but has no impact on translation rates in retinal degeneration. **a** Representive western blots of p-eIF2alpha, eIF2alpha and beta actin in C57BL/6J, *rd16* and *rd16 Gadd34*^−/−^ at P15. **b** Histogram depicting the normalized p-eIF2alpha in the three groups at P15. **c** SUNsET analysis showing nascent protein synthesis in the retinas of C57BL/6J (*n* = 3), *rd16* (*n* = 3) and *rd16 Gadd34*^−/−^ (*n* = 3) mice at P15, the anti-puromycin western blot was normalized to the coomassie stained membrane. **d** Graph showing relative protein synthesis of the three groups. Statistical significance denoted by: *p < 0.05, ***p < 0.005. a.u. arbitrary units, kD kilo Daltons

We previously found an activated ISR in the retinas of *rd16* mice at P15 and P20 as shown by elevations in markers including p-eIF2α, ATF4, and C/EBP homologous protein (CHOP). *rd16* is a mouse model with a rapidly degenerating retina due a spontaneous deletion in the *Cep290* gene, the most frequently mutated gene in Leber congenital amaurosis. Interestingly, GADD34 was only upregulated at P20. Activation of the ISR coincided with a decline in translation rates in *rd16* mice at P15^1^. To assess whether the phosphorylation status of eIF2α plays a role in retinal degeneration, we generated *rd16 Gadd34*^−/−^ mice. As expected, when compared to retinas of *rd16* mice, *rd16 Gadd34*^−/−^ mice displayed a significant elevation (62%) in phosphorylated eIF2α levels (Fig. 1a, b). We next sought whether the elevation in p-eIF2α levels was associated with a change in levels of nascent protein synthesis. Protein synthesis rates were detected using the Surface Sensing of Translation (SUnSET) method^21^. Our adaptation of the SUnSET method to detect nascent peptide synthesis in the retina was described previously^1^. To our surprise, the drastic elevation in p-eIF2α levels did not correspond to a significant change in protein synthesis rates (Fig. 1c, d).

**Fig. 2:**
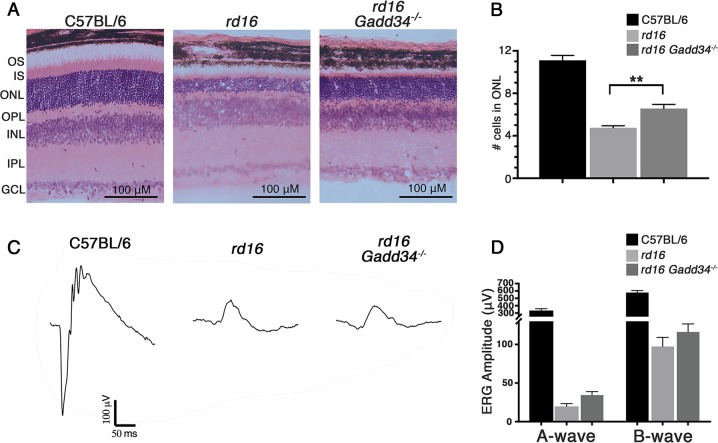
Knocking out *Gadd34* delays retinal degeneration. **a** Representative images of H&E stained sections at P18. **b** Graph depicting mean number of nuclei in the ONL of C57BL/6J (*n* = 3), *rd16* (*n* = 7) and *rd16 Gadd34*^−/−^ (*n* = 10) as counted by a masked investigator. ONL from 400 μM-wide micrographs were counted. **c** Averaged ERG waveforms of dark-adapted mice following a 10 dB flash. **d** Histogram showing average a- and b- wave ERG amplitudes of C57BL/6J (*n* = 4), *rd16* (*n* = 6) and *rd16 Gadd34*^−/−^ (*n* = 10) mice at P17. Statistical significance denoted by: **p < 0.01. OS outer segments, IS inner segments, ONL outer nuclear layer, OPL outer plexiform layer, INL inner nuclear layer, IPL inner plexiform layer, GCL ganglion cell layer

**Fig. 3:**
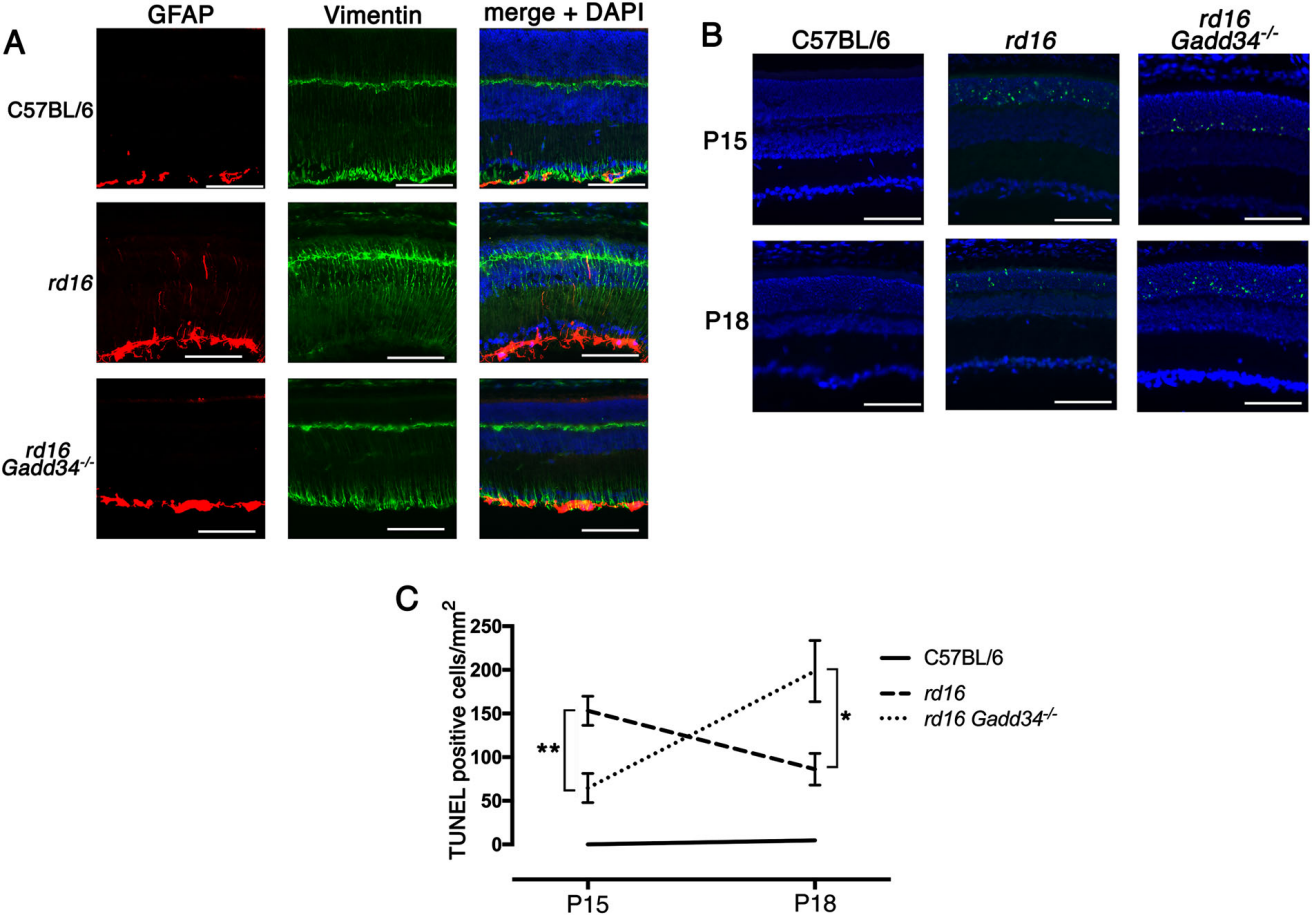
Knocking out *Gadd34* reduces Muller cell gliosis and photoreceptor cell death in retinal degeneration. **a** Muller cell gliosis in retinal sections detected with antibodies against GFAP (red) and vimentin (green) at P18; blue- DAPI. **b** TUNEL staining on retinal sections at P15 and P20; green- TUNEL, blue-DAPI. **c**) Graph showing TUNEL analysis of the three groups (*n* = 3 for C57BL/6J and *rd16*, *n* = 4 for *rd16 Gadd34-/-*) at P15 and P20. Scale bars are equal to 100 µM. Statistical significance denoted by: *p < 0.05, **p < 0.01. ONL outer nuclear layer, INL inner nuclear layer, GCL ganglion cell layer

To determine whether knocking out *Gadd34* had any impact on retinal degeneration, we first counted photoreceptor nuclei in the outer nuclear layer (ONL). To our surprise, we found that *rd16 Gadd34*^−/−^ mice had significantly more nuclei in their ONL at P18 than *rd16* mice (Fig. 2a, b). However, there was no significant difference in electroretinography (ERG) amplitudes between the two groups (Fig. 2c, d), which could be due to non-uniform protection of degenerating photoreceptors across the retina, which is lost when ERG responses of the entire retina are averaged as done in our experiment. The rapid rate of retinal degeneration exhibited by these mice^1,22–26^, and the fact that they do not develop normal outer segments^22,27^, makes them particularly challenging to rescue. There was also not a significant difference in ERG amplitudes between C57BL/6J and *Gadd34*^−/−^ mice at P25 (Fig. S1). We then assessed Müller cell gliosis in these groups by staining retinal cryosections with antibody against glial fibrillary acidic protein (GFAP) and vimentin. GFAP and vimentin are intermediate filament forming proteins. GFAP is highly expressed in gliotic Müller cells and vimentin is expressed in all Müller glia, giving us a way to differentiate between Müller cells and astrocytes^28^. We found that while *rd16* mice had apparent radial GFAP branching corresponding to Müller cells, GFAP staining was largely limited to the inner limiting membrane, and therefore astrocytes, in *rd16 Gadd34*^−/−^ and C57BL/6 mice (Fig. 3a). This suggests that at P18, *rd16 Gadd34*^−/−^ mice demonstrate less Müller cell gliosis than *rd16* mice. We then evaluated cell death in the ONL in these groups by TUNEL analysis. At P15, we found that the retinas of *rd16* mice had significantly less apoptotic cell death than the retinas of *rd16 Gadd34*^−/−^ mice (Fig. 3b, c). At P18 however, *rd16 Gadd34*^−/−^ had significantly more cell death than *rd16*, indicating that apoptosis is delayed in the ONL of *rd16 Gadd34*^−/−^ mice.

### Lowering p-eIF2α levels does not restore protein synthesis and does not delay retinal degeneration

**Fig. 4:**
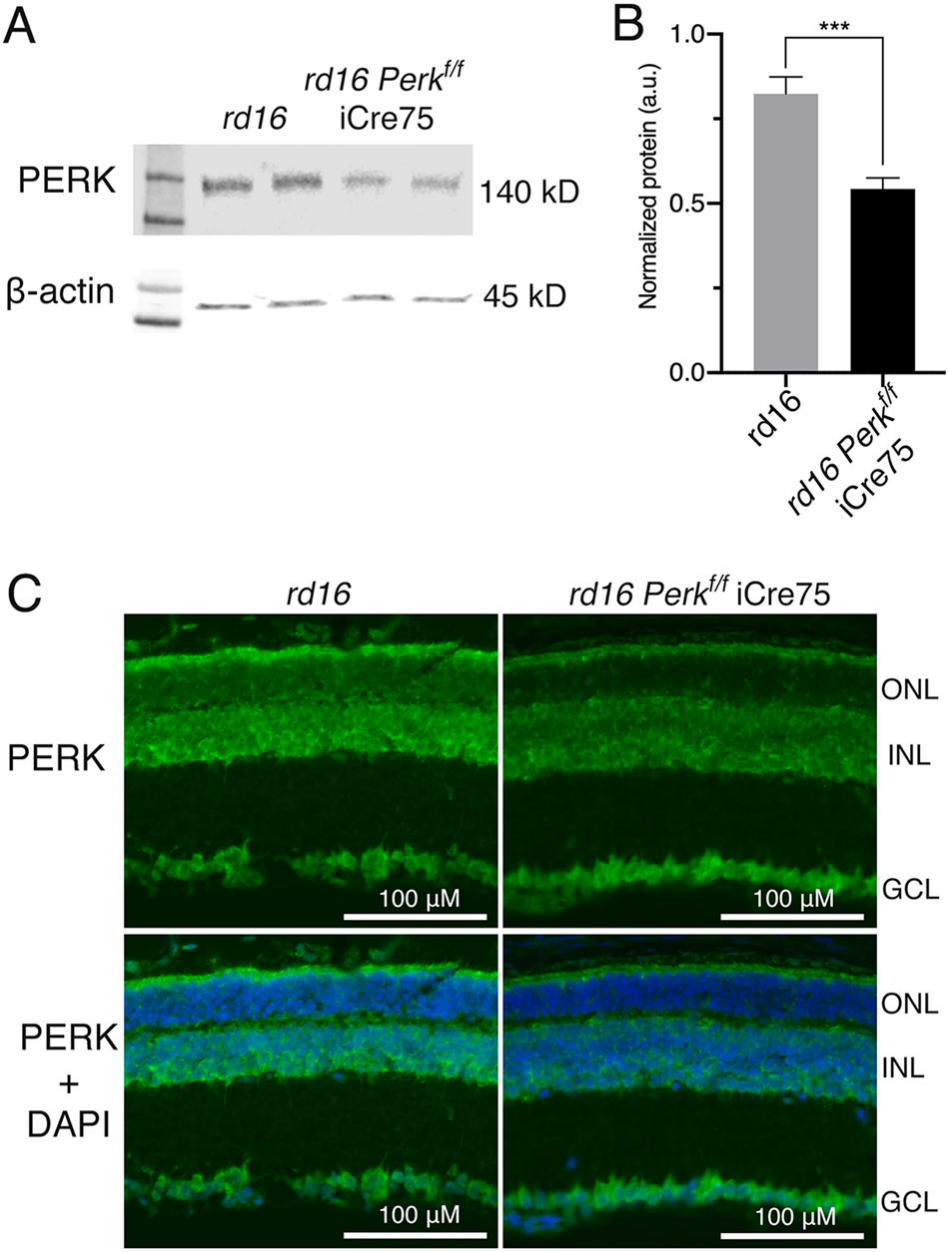
Conditional *Perk* knockout in photoreceptors. **a** Representative western blots of PERK and β-actin in retinal protein lysates isolated from *rd16* and *rd16 Perk*^*f/f*^ iCre75 at P15. **b** Graph showing normalized relative density of PERK in total *rd16* (*n* = 4) and *rd16 Perk*^*f/f*^ iCre75 (*n* = 10) retinal lysates. **c** Immunohistochemistry of PERK in P18 retinal sections. Statistical significance denoted by: ***p < 0.005. a.u. arbitrary units, kD kilo Daltons, ONL outer nuclear layer, OPL outer plexiform layer, INL inner nuclear layer, IPL inner plexiform layer, GCL ganglion cell layer

**Fig. 5:**
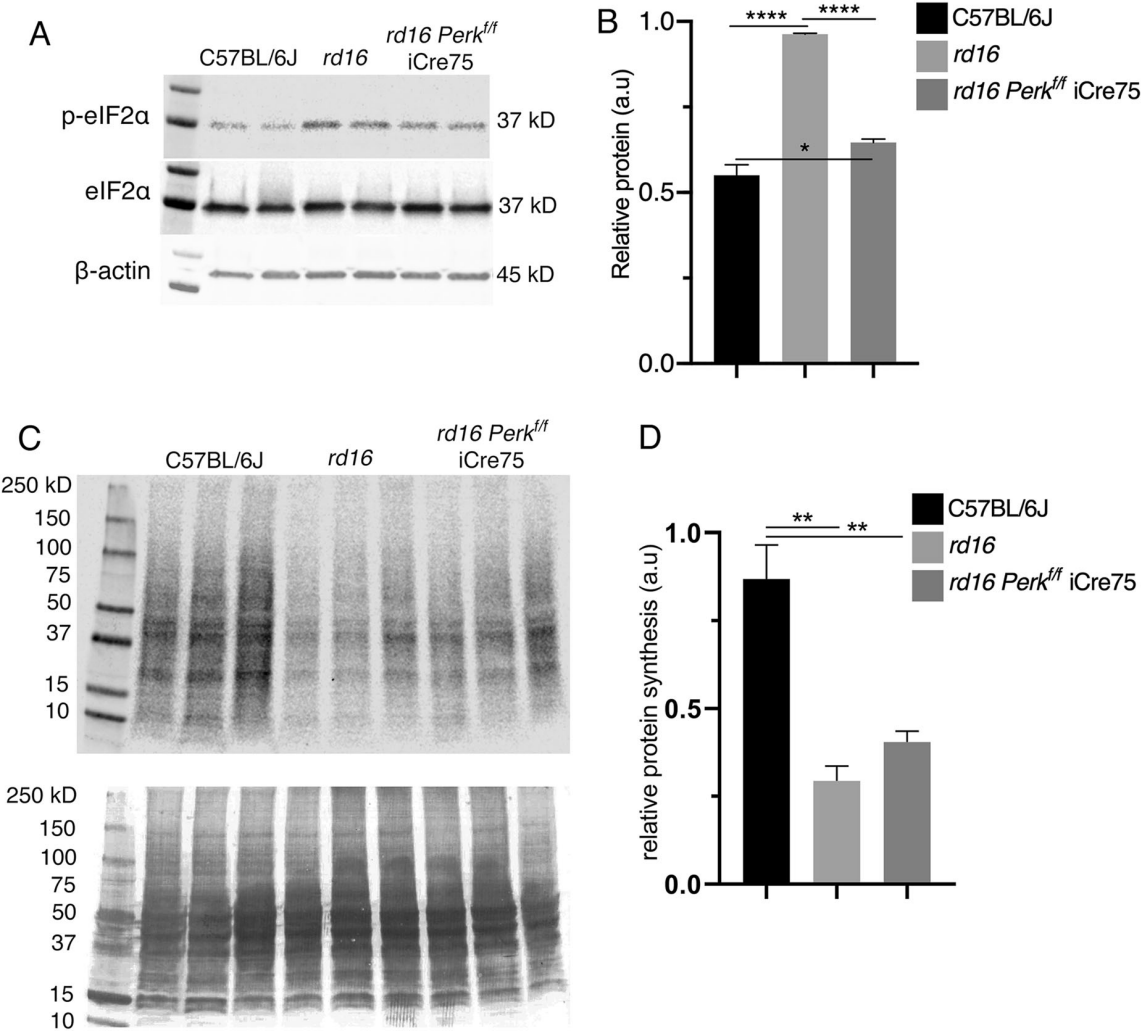
Conditionally knocking out *Perk* in degenerating photoreceptors lowers p-eIF2α levels but has no significant impact on protein synthesis rates. **a** Representative western blots of p-eIF2α, eIF2α and β-actin in retinal protein lysates isolated from *rd16* (*n* = 3) and *rd16* (*n* = 3) *Perk*^*f/f*^ iCre75 (*n* = 3) at P15. **b** Histogram showing normalized p-eIF2α in the three groups at P15. **c** SUNsET analysis showing nascent protein synthesis in the retinas of C57BL/6J (*n* = 3), *rd16* (*n* = 3) and *rd16 Perk*^*f/f*^ iCre75 (*n* = 3) mice at P15, the puromycin western blot was normalized to the coomassie stained membrane. **d** Graph showing relative protein synthesis of the three groups. Statistical significance denoted by: *p < 0.05, **p < 0.01, ***p < 0.005, ****p < 0.001. a.u. arbitrary units, kD kilo Daltons

**Fig. 6:**
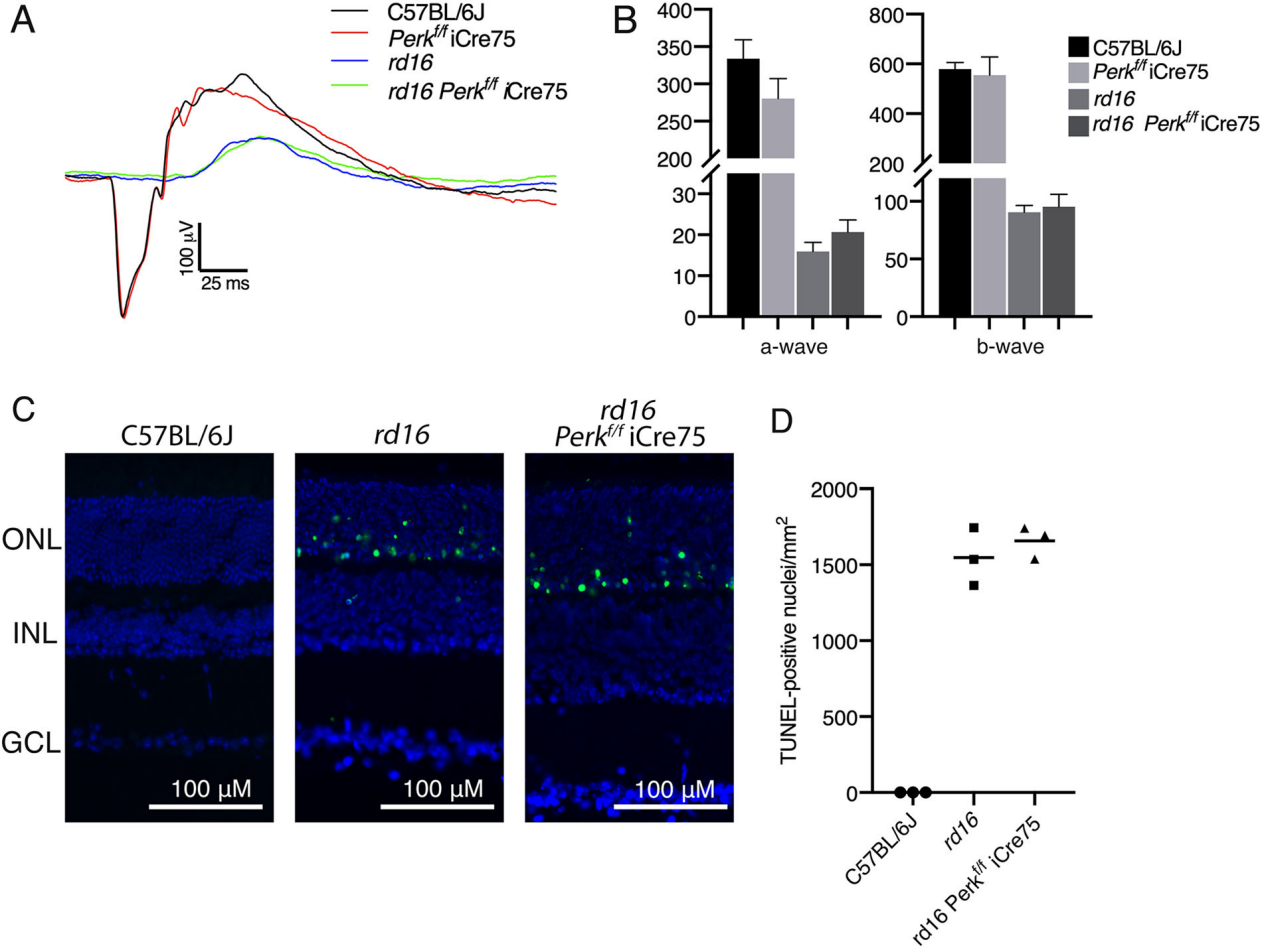
Lowering p-eIF2α levels has no impact on retinal degeneration. **a** Averaged dark-adapted ERG waveforms at P17. **b** Graphs showing means of a- and b- wave ERG amplitudes of C57BL/6J (*n* = 7), *Perk*^*f/f*^ iCre75 (*n* = 6), *rd16* (*n* = 5) and *rd16 Perk*^*f/f*^ iCre75 (*n* = 7) mice. **c** Representative TUNEL-stained sections. green-TUNEL, blue- DAPI. **d** Graph showing TUNEL analysis of C57BL/6J (*n* = 3), *rd16* (*n* = 3) and *rd16 Perk*^*f/f*^ iCre75 (*n* = 3) at P15. ONL outer nuclear layer, INL inner nuclear layer, GCL ganglion cell layer

Since higher p-eIF2α levels in mice lacking *Gadd34* were associated with a delay in retinal degeneration in *rd16* mice, we next hypothesized that lowering p-eIF2α levels by targeting PERK would worsen retinal degeneration. We previously proposed that the majority of protein synthesis attenuation seen in the retina during RD may not be due to eIF2α regulation^1^. In that study, we pharmacologically inhibited PERK in *rd16* mice and discovered that while p-eIF2α levels decreased by about 50% in treated mice, protein synthesis only recovered by ~17%. Since PERK inhibiting compounds are non-specific^29^ and highly toxic^30^, we decided to implement a genetic model to study the role of PERK and eIF2α in RD. To that end, we generated *Perk*^*f/f*^ iCre75 and *rd16 Perk*^*f/f*^ iCre75 mice. iCre75 mice house a *Cre* transgene under the control of a mouse opsin promoter, which gives rise to rod photoreceptor specific knockout of floxed genes^31^, therefore enabling us to study the role of the PERK in photoreceptors. We first assessed PERK levels in the retinas of *rd16* and *rd16 Perk*^*f/f*^ iCre75 mice by western blot analysis and IHC. Proteins were extracted from whole retina lysates and subjected to western blot analysis. As expected, PERK levels in total retina protein lysates were significantly lower (~34%) in *rd16 Perk*^*f/f*^ iCre75 than *rd16* mice (Fig. 4a, b). In addition, compared to *rd16*, there was a prominent reduction in PERK immunofluorescence in both the cell bodies surrounding the photoreceptor cell nuclei in the ONL and inner segments, of *rd16 Perk*^*f/f*^ iCre75 mice (Fig. 4c), whereas the immunofluorescence of the INL and GCL is more consistent between the two groups. Importantly, the immunofluorescent regions in the micrographs are consistent with ER localization^32^. We next assessed whether this decrease in PERK levels corresponded to a reduction in levels of p-eIF2α in *rd16 Perk*^*f/f*^ iCre75 mice. To that end, p-eIF2α levels were assessed in retinal lysates isolated from C57BL/6J, *rd16* and *rd16 Perk*^*f/f*^ iCre75 mice at P15. As expected, the retinas of *rd16* mice exhibited a significant increase in p-eIF2α levels compared to C57BL/6J (Fig. 5a, b). However, *rd16 Perk*^*f/f*^ iCre75 retinas had significantly lower p-eIF2α levels (~32%) than the *rd16* mice, demonstrating that although levels were still significantly higher (~17%) in the retinas of *rd16 Perk*^*f/f*^ iCre75 mice than C57BL/6J mice, loss of *Perk* was associated with an almost complete normalization in eIF2α phosphorylation (Fig. 5b). We next assessed whether the decrease in p-eIF2α levels was associated with a change in protein synthesis rates. Interestingly, the lower p-eIF2α levels in *rd16 Perk*^*f/f*^ iCre75 mice were not associated with a significant increase in protein synthesis rates (Fig. 5c, d). Together with the translation data from *rd16 Gadd34*^−/−^ mice and our previous report showing that PERK inhibition only marginally restores protein synthesis rates in the retina^1^, these results demonstrate that eIF2α regulation is not the primary means by which degenerating retinas control protein synthesis rates. Nevertheless, a group previously reported that the phosphorylation state of eIF2α may play a role in retinal degeneration^5^. Therefore, we next compared the retinal viability of these three groups. We assessed the retinal function of these groups using scotopic ERG at P17. We did not detect a significant difference in a- or b-wave amplitude between *rd16* and *rd16 Perk*^*f/f*^ iCre75 (Fig. 6a, b). Importantly, ERG amplitudes of C57BL/6J mice and *Perk*^*f/f*^ iCre75 mice were not significantly different at this time point. Further, there was also no detectable difference in ERG amplitudes between C57BL/6J mice and *Perk*^*f/f*^ iCre75 mice at P60 (Fig. S2). We then assessed apoptotic photoreceptor cell death in the ONL at P15 by TUNEL analysis. To our surprise, we found that there was not a significant difference in the number of TUNEL-positive nuclei between between *rd16* and *rd16 Perk*^*f/f*^ iCre75 (Fig. 6c, d).

### Loss of *Gadd34* is associated with AKT activation in degenerating retinas

**Fig. 7:**
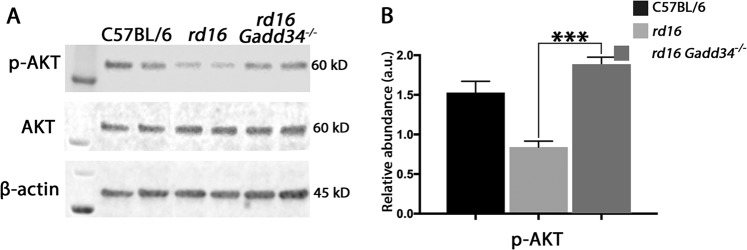
Knocking out *Gadd34* is associated with an elevation in AKT activation in degenerating retinas. **a** Western blots of p-AKT, AKT and β-actin. **b** Analysis of normalized AKT phosphorylation in the retinal lysates isolated from C57BL/6J (*n* = 5), *rd16* (*n* = 4) and *rd16 Gadd34*^−/−^ (*n* = 5) mice at P15. Statistical significance denoted by: ***p < 0.005. a.u. arbitrary units, kD kilo Daltons

Since knocking out *Gadd34* resulted in a significant increase in p-eIF2α levels in *rd16* mice that correlated with a delay in retinal degeneration, we expected the loss of PERK in photoreceptors to have the opposite effect due to lower p-eIF2α levels. This made us wonder if something else could be occurring in the retinas of *rd16 Gadd34*^−/−^ mice. Although eIF2α does not have any known function outside of protein synthesis regulation, GADD34 has a multitude of proposed functions outside of eIF2α regulation^13–15,33^. One particular function of GADD34 is its well-documented role as a pro-apoptotic protein^14,16,34^. One pro-apoptotic duty of GADD34 is its inhibition of AKT activation^14,16,34^. We previously reported that *rd16* mice have diminished levels of p-AKT at P15^1^. To determine whether the delay of RD in *rd16 Gadd34*^−/−^ mice could have be due to enhanced AKT activation, we assessed levels of p-AKT (Ser473) in the retinas of C57BL/6, *rd16* and *rd16 Gadd34*^−/−^ mice at P15. Interestingly, compared to *rd16* mice, the retinas *rd16 Gadd34-/-* mice had significantly elevated levels of p-AKT (Fig. 7a, b), which may indicate that the knockout of *Gadd34* improves photoreceptor viability by stimulating AKT signaling rather than through regulation of eIF2α.

## Discussion

Multiple reports have proposed regulating the ISR to ameliorate retinal degeneration^5,35–37^. In the present study, we provide evidence suggesting that regulation of eIF2α alone may not be sufficient to significantly change protein synthesis rates or impact retinal cell viability. In fact, reducing p-eIF2α levels by knocking out *Perk* in retinal photoreceptors had no impact on photoreceptor survival in *rd16* mice. However, an elevation in p-eIF2α levels in the retinas of *rd16 Gadd34*^−/−^ mice was associated with a delay in photoreceptor cell death even in the absence of a change in the levels of protein synthesis. There are no known functions of eIF2α outside of protein synthesis regulation, but there are many other functions of GADD34^13–16,33^. Therefore, it is likely that the delay in RD observed *rd16 Gadd34*^−/−^ is due to the loss of GADD34 rather than the change in p-eIF2α levels. In fact, there have been a number of studies that have implicated GADD34 in the induction of apoptosis^14,16,34^. It is possible that GADD34 ablation could selectively regulate translation of anti-apoptotic proteins in degenerating retinas, which could be due to a 5′-cap independent, or internal ribosome entry site (IRES) mechanism. One well-characterized pro-apoptotic role of GADD34 is its ability to inhibit membrane-translocation of AKT and its subsequent activation^14^. AKT has a multitude of pro-survival functions as it directly phosphorylates a number of pro-apoptotic and apoptotic proteins. We showed that the retinas of *rd16* mice lacking *Gadd34* experienced a delay in photoreceptor cell death that coincides with an elevation in AKT phosphorylation, which is consistent with the reported role of Gadd34 in AKT inactivation.

The viability of *Gadd34*^−/−^ mice has been reported previously^38–40^, so there was little surprise that mice lacking GADD34 exhibited normal ERG amplitudes. However, we found it particularly surprising that PERK does not seem to be needed for photoreceptors. Whereas *Gadd34*^−/−^ was knocked out systemically in our study, we used a Cre-recombinase driven by a mouse opsin promoter to knock out *Perk* in photoreceptors. Using a conditional knockout of *Perk* allowed us to study the role of PERK in photoreceptors without the issue of pancreatic atrophy, however, the iCre75 transgenic mouse does not show recombination around loxP sites until P7 and recombination persists until as late as P18^31^. Therefore, whether PERK is important for photoreceptor development could not be assessed in the present study.

The results of this study suggest that regulating eIF2α phosphorylation may not be enough to delay apoptosis. However, knocking out GADD34 resulted in a delay in retinal degeneration but had no impact on retinal function as assessed by ERG. This could be due to the *rd16* mouse model having a photoreceptor ciliopathy^22,23^, as these animals do not form normal outer segments^22,27^. This makes *rd16* mice particularly challenging to rescue. It would perhaps be better to assess the role of GADD34 in another animal model such as one that does not degenerate so rapidly or more directly assess the role of GADD34 in *rd16* retinas under simultaneous inhibition of p-eIF2α by ISRIB compound. The role of PERK under chronic ISR activation should also be verified by using other animal models, even though this kinase appears to be redundant in wild type retinas. Another possible reason that p-eIF2α modulation may not affect protein synthesis is the eIF2 guanine exchange factor, eIF2B. eIF2 is the translation initiation factor that brings the initiator methionine to the pre-initiation complex. eIF2B is the rate-limiting step regulating formation of the pre-initiation complex. It is possible that in *rd16* retinas, eIF2B could have saturated affinity of binding to p-eIF2α, thus, limiting the therapeutic targeting of p-eIF2α. In addition, if eIF2 is truly only regulating a small portion of protein synthesis in degenerating retinas, some other translation factor-initiator tRNA complex must have high activity in the retina.

Recently, research conducted in animal models of PD, AD and tauopathies have demonstrated the therapeutic effect from targeting the PERK arm of the UPR including eIF2α, GADD34 and PERK^18,41–44^. Through modulation of these factors, these studies have shown improvement of neuronal function and viability. The results of the current study however, aimed at validating the PERK UPR arm as a therapeutic target and suggested that this signaling may not be the most efficient strategy to protect neuronal cell death. Previously, we have reported diminished levels of AKT → mTOR → 4E-BP1 signaling in *rd16* retinas^1^. Therefore, investigation of this signaling will significantly enhance our molecular understanding of translational control in degenerating retinas.

## Materials and methods

### Animals

All animal experiments followed a protocol (IACUC#131109793) approved by the University of Alabama at Birmingham Institutional Animal Care and Use Committee (IACUC), and conformed to guidelines set by the Association of Research in Vision Science and Ophthalmology. BXD24/TyJ-*Cep290*^*rd16*^/J (000031, *rd16*), *Eif2ak3*^tm1.2Drc^/J (023066, *Perk*^*f/f*^)and C57BL/6J (000664) mice were obtained from Jackson Laboratory (Bar Harbor, ME). *Gadd34* KO mice were provided by Dr. David Ron and have been previously described^45^. iCre75 mice have been described^31^. These mice were crossed with *rd16* mice to yield the genotypes mentioned in this article. Genotyping of each of these mice has been mentioned previously. Mice were housed in a facility with a 12-hour light/dark cycle. Mice had free access to a standard diet and water. At the time points specified in the following sections and text body, mice were euthanized by CO_2_ asphyxiation followed by cervical dislocation.

### Western Blotting

For western blot analyses, mouse retinas were dissected and homogenized in lysis buffer (150 mM NaCl, 1.0% Triton X-100, 0.5% sodium deoxycholate, 0.1% SDS, and 50 mM Tris pH 8.0). Protein concentrations were estimated by the Bio-Rad protein assay (5000001, Hercules, CA, USA). Protein lysate (40–60 μg) were separated by SDS-PAGE and transferred to a PVDF membrane for immunoblotting. Primary antibodies specific for phospho-eIF2α (p-S51, 3398, rabbit, lot: 6), eIF2α (9722, rabbit, lot: 15), p-AKT (p-S473, 4060, rabbit, lot: 14), AKT (4691, rabbit, lot: 20) were purchased from Cell Signaling Technologies (Danvers, MA, USA). The anti-β-actin primary antibody (A2228, mouse) was purchased from Sigma Aldrich (St louis, MO, USA). Secondary antibodies (WesternSure HRP goat anti-mouse IgG: 926–80010, WesternSure HRP goat anti-rabbit IgG: 926–80011, and IRDye 800CW Goat anti-mouse: 925–32210) were purchased from LI-COR (Lincoln, NE, USA). Probed membranes were imaged on a LICOR Fc imaging system. Relative band densities were measured using ImageJ software. Westerns were normalized to β-actin. Phosphorylated proteins were normalized to their non-phosphorylated counterparts.

### Histology and immunohistochemistry

Eyes were enucleated at P15 or P18 and washed in phosphate buffered saline. Eyes were then placed in 4% paraformaldehyde (diluted in PBS from 16% paraformaldehyde, electron microscopy sciences 15710; Hatfield, PA) for 30 min at room temperature. After 30 min, a needle (33G) was inserted at the limbus to create a small hole for 4% paraformaldehyde to enter. These eyes were then fixed in 4% paraformaldehyde at 4 °C for 4 h or overnight, depending on the assay for which they were used (4 h: IHC, H&E. overnight: TUNEL). Fixed eyes were washed with PBS and immersed in 30% sucrose for at least 1 h. Eyes were then removed from sucrose and embedded in Tissue-Tek O.C.T. compound (VWR: 25608–930) and kept at −80 °C for 1 h. Cryomolds were then equilibrated to the temperature inside (−21 °C) the cryostat sectioning system (Leica CM 1510S; Leica, Buffalo Grove, IL, USA). 12 μM eye sections were cut using a cryostat tissue sectioning system. Sections were stained with hematoxylin and eosin (Electron Microscopy Sciences: 26754–1A, 26762–01). A masked investigator counted the number of rows of nuclei in the ONL in 400 μM-wide micrographs. Anti-GFAP (G3893) was purchased from Sigma-Aldrich and anti-vimentin (5741) was purchased from Cell Signaling Technologies. The antibody against PERK (sc-377400 AF488) was purchased from Santa Cruz Biotechnology (Dallas, TX, USA). Terminal deoxynucleotidyl transferase (TdT) dUTP Nick-End Labeling (TUNEL)-staining (Click-it plus TUNEL assay, ThermoFisher scientific, C10617) was performed on retinal sections following instructions from the manufacture. Sections were counterstained with DAPI (Vector Laboratories, H-1200). TUNEL-positive nuclei were counted automatically using the RETINA analysis toolkit for ImageJ. Images of sections were acquired using a Zeiss Axio-plan2 fluorescent microscope.

### Analysis of nascent protein synthesis

The SUnSET method has been described previously^1,21^. For in vivo analysis of nascent peptide synthesis, mice were intraperitoneally injected with puromycin (puromycin dihydrochloride; Santa Cruz Biotechnology, CAS 58–58–2) at a dosage of 0.04 μmol/g body mass. Retinas were harvested 30 min after the puromycin injection. Proteins were then extracted in RIPA buffer and protein concentrations were estimated as stated above in the western blotting section. Between 40 and 60 μg of protein were separated by SDS-PAGE and then transferred to a PVDF membrane. Membranes were then incubated with an antibody specific to Puromycin (MABE343, mouse, lot: 2861354), which was purchased from EMD Millipore. A secondary antibody specific to IgG2a (goat anti-mouse peroxidase affinipure IgG, Fcγ Subclass 2a Specific: 115–035–206, Jackson Immuno Research Laboratories Inc; West Grove, PA, USA) was used to prevent detection of endogenous immunoglobulin heavy and light chains. As a loading control, membranes were stained with coomassie blue R-250 (Bio-Rad: #1610436). After anti-puromycin immunoblotting, membranes were washed with distilled water and then incubated in coomassie staining solution for 60 s. Membranes were then de-stained (ethanol/acetic/water; 5:1:4 proportion) for 10–30 min and air dried before scanning on a Kyocera Taskalfa copier. Relative densities of entire lanes were measured using ImageJ software. Puromycin densities were normalized to their corresponding coomassie densities.

### Electroretinography

Mice were dark adapted overnight. Electroretinography was conducted using a LKC BIGSHOT ERG instrument. Briefly, mice anesthetized with ketamine and xylazine and then their eyes were dilated. Once dilated, the animals were placed on the instrument. Mice were then exposed to 5 flashes of 25 cd.s/m^2^ in 45 s intervals. ERG waveforms were then analyzed using LKC EM software.

### Statistics

Student *t* test was used to compare two groups and ANOVA was carried on comparisons made between three or more groups. All statistics were performed using Graphpad Prism 8 software.

## Supplementary information

Supplemental Figure S1

Supplemental Figure S2

Supplementary figure legends
