# Supplementary figures and images for: Delineating the role of eIF2α in retinal degeneration
Source: Cell Death Dis. 2019 May 28;10(6):409. doi: 10.1038/s41419-019-1641-y (PMC6538684; doi:10.1038/s41419-019-1641-y)

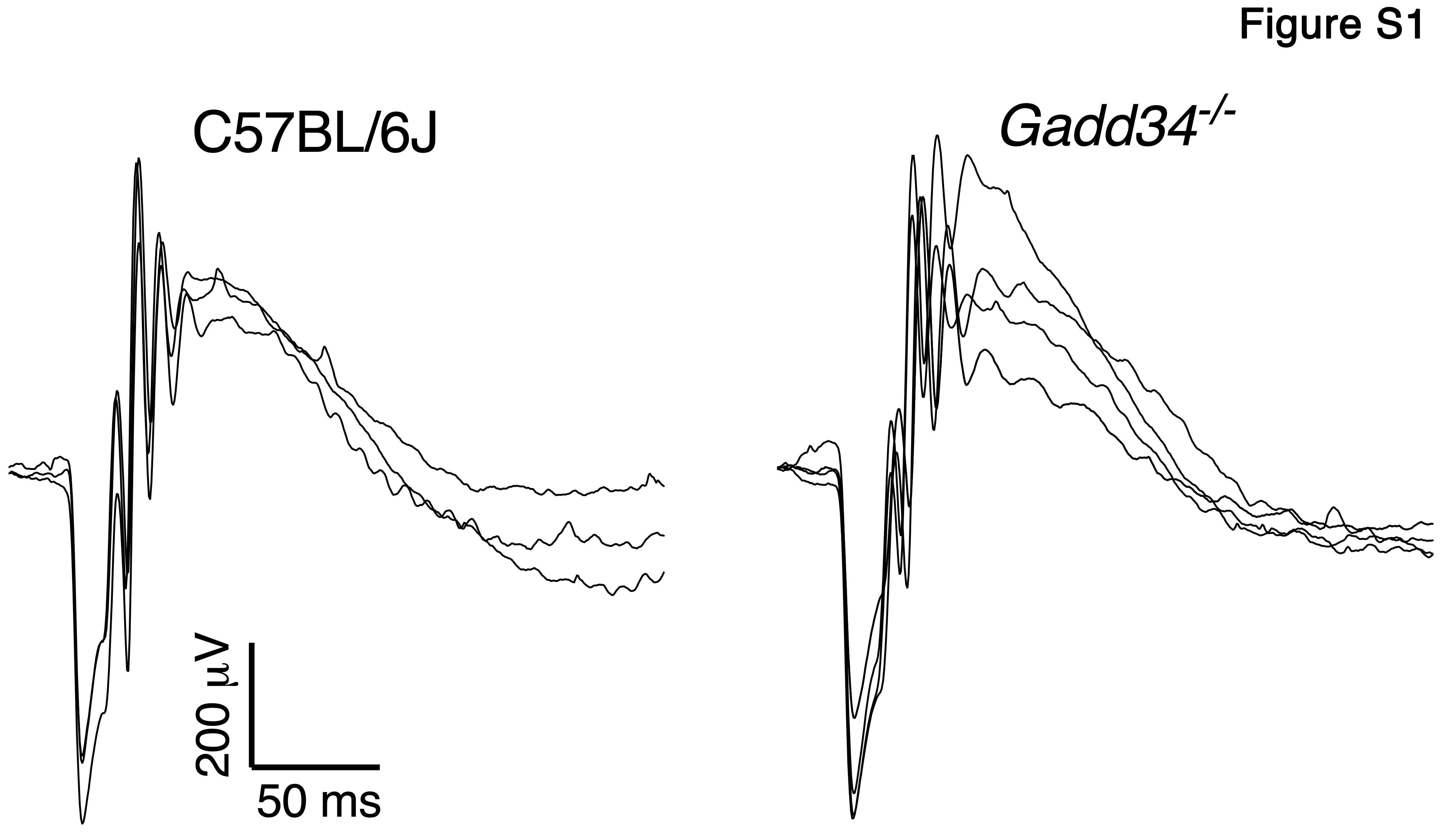

Supplement: Supplementary file 1 — Supplemental Figure S1 [file 41419_2019_1641_MOESM1_ESM.tif]

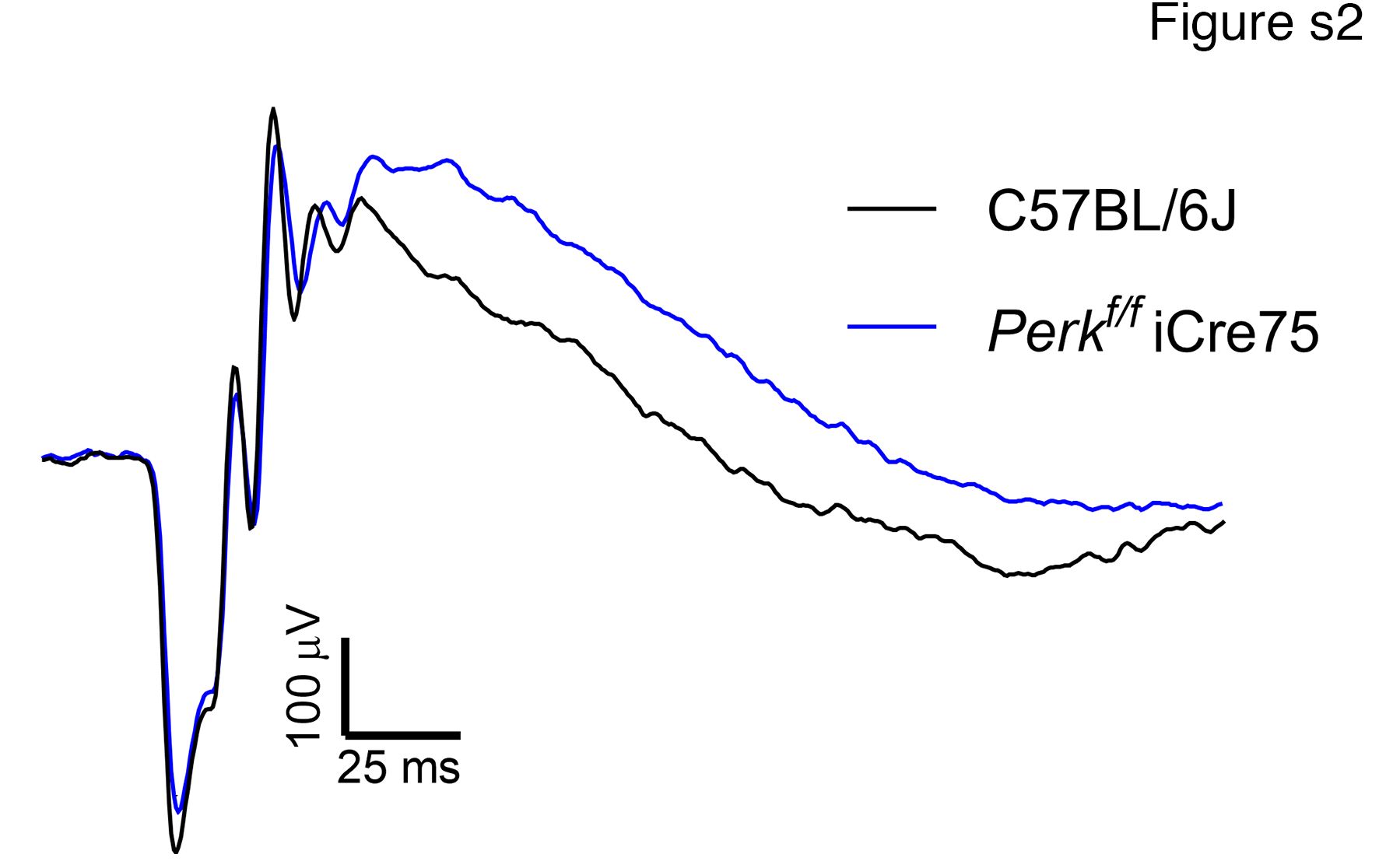

Supplement: Supplementary file 2 — Supplemental Figure S2 [file 41419_2019_1641_MOESM2_ESM.tif]
