# Supplementary material for: Delineating the role of eIF2α in retinal degeneration
Source: Cell Death Dis. 2019 May 28;10(6):409. doi: 10.1038/s41419-019-1641-y (PMC6538684; doi:10.1038/s41419-019-1641-y)
Supplement: Supplementary file 3 — Supplementary figure legends [file 41419_2019_1641_MOESM3_ESM.docx]

**Supplementary Figure Legends**

**Figure S1**- Systemically knocking out *Gadd34* does impact the function of the retina. Individual ERG waveforms of C57BL/6J and *Gadd34^-/-^* mice at P25.

**Figure S2**- Conditionally knocking out *Perk* in photoreceptors does not negatively affect retinal function. ERG waveforms of C57BL/6J and *Perk^f/f^* iCre75 at P60.
